# FHIR-PYrate: a data science friendly Python package to query FHIR servers

**DOI:** 10.1186/s12913-023-09498-1

**Published:** 2023-07-06

**Authors:** René Hosch, Giulia Baldini, Vicky Parmar, Katarzyna Borys, Sven Koitka, Merlin Engelke, Kamyar Arzideh, Moritz Ulrich, Felix Nensa

**Affiliations:** 1 Institute of Interventional and Diagnostic Radiology and Neuroradiology, University Hospital Essen, Hufelandstraße 55, Essen, 45147 Germany; 2 Institute for Artificial Intelligence in Medicine, University Hospital Essen, Girardetstraße 2, Essen, 45131 Germany; 3 Central IT Department, Data Integration Center, University Hospital Essen, Hufelandstraße 55, Essen, 45147 Germany

**Keywords:** Electronic patient record, FHIR, Python, Dataframe, Information extraction, Dicom

## Abstract

**Background:**

We present FHIR-PYrate, a Python package to handle the full clinical data collection and extraction process. The software is to be plugged into a modern hospital domain, where electronic patient records are used to handle the entire patient’s history. Most research institutes follow the same procedures to build study cohorts, but mainly in a non-standardized and repetitive way. As a result, researchers spend time writing boilerplate code, which could be used for more challenging tasks.

**Methods:**

The package can improve and simplify existing processes in the clinical research environment. It collects all needed functionalities into a straightforward interface that can be used to query a FHIR server, download imaging studies and filter clinical documents. The full capacity of the search mechanism of the FHIR REST API is available to the user, leading to a uniform querying process for all resources, thus simplifying the customization of each use case. Additionally, valuable features like parallelization and filtering are included to make it more performant.

**Results:**

As an exemplary practical application, the package can be used to analyze the prognostic significance of routine CT imaging and clinical data in breast cancer with tumor metastases in the lungs. In this example, the initial patient cohort is first collected using ICD-10 codes. For these patients, the survival information is also gathered. Some additional clinical data is retrieved, and CT scans of the thorax are downloaded. Finally, the survival analysis can be computed using a deep learning model with the CT scans, the TNM staging and positivity of relevant markers as input. This process may vary depending on the FHIR server and available clinical data, and can be customized to cover even more use cases.

**Conclusions:**

FHIR-PYrate opens up the possibility to quickly and easily retrieve FHIR data, download image data, and search medical documents for keywords within a Python package. With the demonstrated functionality, FHIR-PYrate opens an easy way to assemble research collectives automatically.

## Introduction

The HL7 (*Heath Level Seven*) FHIR (*Fast Healthcare Interoperability Resources*) standard [1] is steadily developing into the healthcare standard for semantic interoperable data exchange [2]. Clinical routines are not exempt from the digitalization process that the world is currently experiencing [3, 4]. As a consequence, hospitals are evolving and adapting. Many hospitals and healthcare organizations are already using EHRs (*Electronic Health Records*) to store and manage patient health information [5]. The ONC (*Office of the National Coordinator for Health Information Technology*) estimated that in 2021 in the US “nearly 4 in 5 office-based physicians (78%) and nearly all non-federal acute care hospitals (96%) adopted a certified EHR” [6]. EHRs can help to improve the quality and safety of patient care by providing healthcare providers with access to up-to-date and accurate patient information [7–9]. However, EHRs can also create challenges for data exchange between different healthcare systems, as there are often multiple EHR systems in use [10]. To address these challenges, healthcare organizations may use a variety of systems and standards for data exchange, such as HL7 v2 [1], HL7 v3 [1], HL7 CDA (*Clinical Document Architecture*) [11], openEHR [12] or HL7 FHIR [1]. However, FHIR is the newest and most widely used one [1, 2, 13]. The ONC estimated that in 2019 “84% of hospitals and 61% of clinicians adopted and implemented 2015 Edition certified API technology enabled with FHIR” and that the trend is expected to continue [14]. Furthermore, FHIR is not only utilized in clinical practice but is also a subject of ongoing research and development [15–20]. One of the main advantages of FHIR is its uniform structure and reproducibility for all information created within clinical workflows. This strive for standardization is especially needed in the world of hospital data, which includes multiple manufacturers, standards, and ways to store the overall complex clinical data. Because of the considerable success of this standard, manufacturers of medical applications have already started integrating it into their tools. Notable examples are the AI-Pathway Companion [21] and the Azure Health Data Services API (*Application Programming Interface*) [22], but other well-known companies also rely on open interfaces such as FHIR to facilitate interoperability [23, 24].

Together with the popularity of FHIR, machine learning applications and studies in clinical context have also steadily increased [25]. For the year 2022, 13,931 articles can be found on PubMed by searching for results including both "machine learning" or "artificial intelligence" and "healthcare" or "clinical", a number that is higher than any of the previous years. Thus, it is not only important to have a standard for the data format (FHIR), but also to build a uniform way to easily extract data and to transfer them into formats preferred for machine learning. As technology advances, multimodal models (i.e., accepting both images, unstructured text and structured information) are also becoming more and more relevant [26], yielding that FHIR is also going to be part of previously only image-based pipelines.

The paper has the following structure. First, the “Background” section defines and introduces basic FHIR terminology, resources, and components. In addition, an overview of the current number of FHIR resources is given using the example of the University Hospital Essen, comparable tools are discussed and the advantages of FHIR-PYrate are explained. Subsequently, all important components and functions of the package are presented in the "Implementation" section. Subsequently, in the "Results" section, an application-oriented example of a cohort compilation of metastatic breast cancer patients with the help of FHIR-PYrate is presented. Finally, in the "Conclusion" and "Discussion" sections advantages of the tool are described and a final conclusion about the capabilities of the package is drawn.

## Background

Since many FHIR-related terms are used in this paper, the FHIR specification will be introduced first. The FHIR standard builds an abstraction layer on top of one or many databases, where healthcare-related data are stored. This abstraction provides an intuitive and semantically interoperable data exchange, where every single entity can be described as one object or, in FHIR terms, one resource. In general, FHIR (in version R4B) includes 140 different resource definitions [27]. Based on these definitions, all types of hospital data collected within the clinical routine can be categorized and stored in a structured way. This includes, for example, patient data, image data, laboratory values, ward assignments, procedures, medications, medical documents, and many more. In the following, we will briefly describe four resources: Patient, DiagnosticReport, ImagingStudy, Observation, and Bundle.

Almost every data point created within the clinical routine is associated with a patient. Thus, the Patient resource is often the basis for creating and collecting cohorts. This resource contains relevant data such as gender, age, date of birth, or addresses, and also information about the vital status. Attributes can, in turn, have further nested attributes to describe a specific setting more precisely. For example, the patient's multiple addresses could be represented by a list with attributes such as city, address line, and postal code. This yields that a resource might have an arbitrarily complex structure but still conform to the standard. Another essential resource is DiagnosticReport, which includes medical documents such as radiological findings, pathological findings, doctor's letters, and many more. In addition, FHIR offers the possibility to map all imaging data in the form of the ImagingStudy resource. This means that all types of imaging modalities such as CT (*Computed Tomography*), MRI (*Magnetic Resonance Imaging*), ultrasound, or X-ray are available in a structured way, and entire studies, including the corresponding series, can be mapped. It is important to note though, that the image payload, in particular the pixel data, is not contained in the ImagingStudy resource but normally provided via references to a specialized service like a DICOM (*Digital Imaging and Communications in Medicine*) or DICOMweb server. Finally, Observation is a versatile resource, which offers a structure for any measured value, such as laboratory values but also short text like clinical notes. All resources in FHIR have their own unique ID but can be combined per patient via the associated patient ID. The communication with the FHIR server is carried out with a standardized REST (*REpresentational State Transfer*) protocol [1], which allows for creating, reading, replacing, modifying or deleting data. The server returns a FHIR Bundle, which is a collection of FHIR resources matching the search parameters with additional metadata related to the search results. Other uses of Bundles include transactionally modifying multiple resources, or grouping multiple related resources into a coherent view. Thus, FHIR provides the basic framework to retrieve clinical data in a structured way. While the FHIR specification itself allows many degrees of freedom with respect to the type and cardinality of the attributes of FHIR resources, so-called FHIR profiles are used to concretize the semantically interoperable exchange data.

**Fig. 1:**
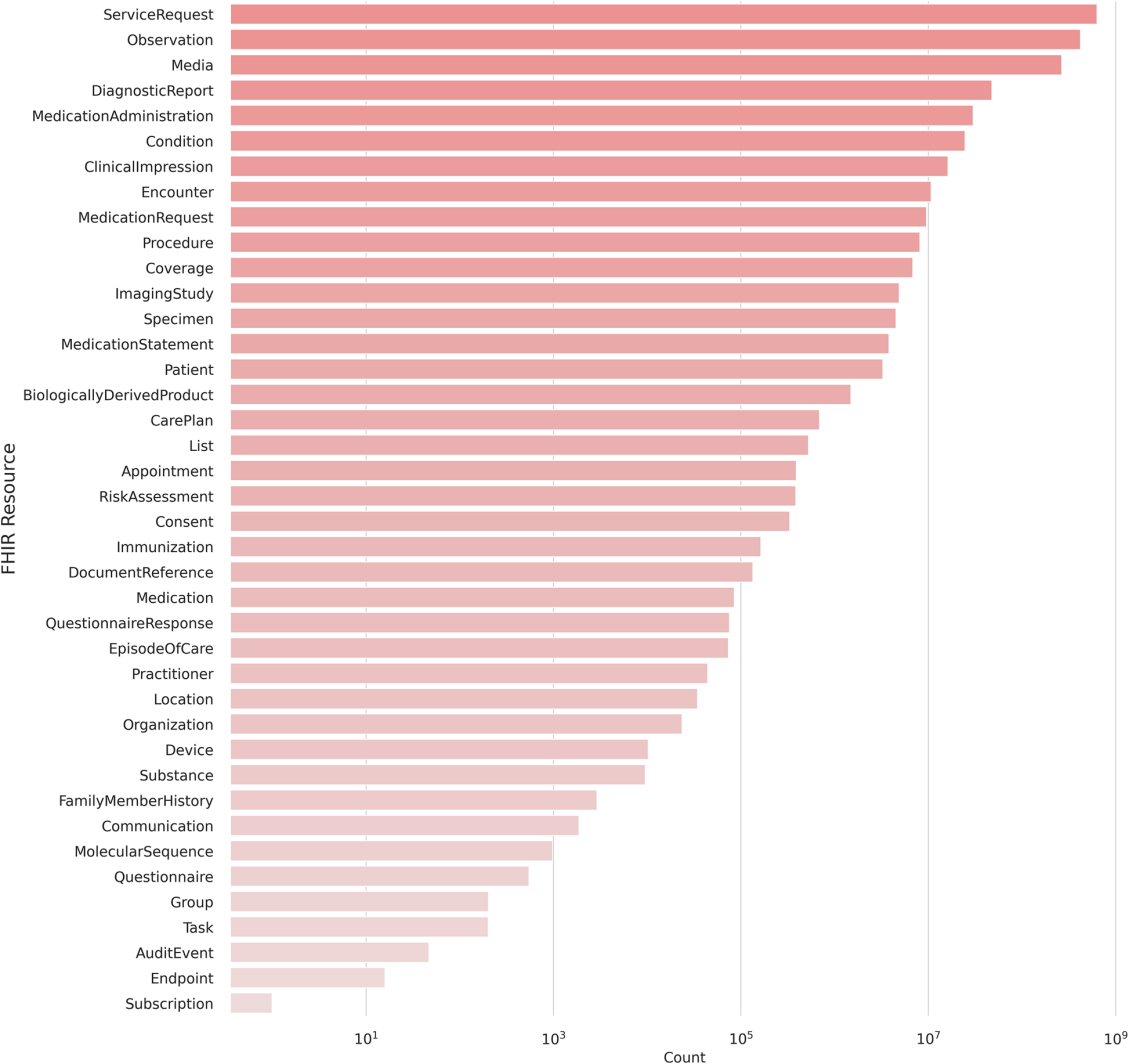
Resource statistics at our institution. The plot shows the distribution of the 1,498,863,142 resources present on our FHIR server as of 2023–03-13. The most common resource is ServiceRequest, which is used for records of requests for procedures, diagnostics, or any other service. The second most common is Observation, which stores measurements like lab values, vital parameters but also small texts like clinical notes

At our institution, the FHIR servers store over 1 billion FHIR resources (see Fig. 1). These encompass approximately 4.6 million imaging studies, 45 million fully indexed clinical text documents, and 389 million clinical observations from more than 3 million patients, as presented in Fig. 1. Thus, it is particularly relevant to have a fast, simple, parallelizable tool that lets the client leverage the capabilities of the FHIR REST API and handle the most expensive steps server-side.

Although this standard has excellent capabilities for exchanging data in a semantically interoperable manner, it is not optimal for statistical data analysis or machine learning in its primary form. Recent studies and tools use preprocessing on FHIR resources to limit and select the input for statistical analysis [28–30]. However, there also exist deep learning pipelines that use preprocessing and tokenization of FHIR resources to make predictions [31]. Nonetheless, this is still not feasible for statistical software or for purely clinical studies. FHIR resources are typically nested, and their degree of complexity is usually unbounded, while data structures used for data science (e.g., pandas’ *DataFrames* [32, 33] for Python, data frames in R) are often tabular data. This mismatch calls for a fast, simple, and easy-to-use tool that acts as a bridge between FHIR and the data science world. We aim to build a simple Python abstraction utilizing the existing FHIR REST API functionalities, giving the user complete control over the queries and providing results as pandas *DataFrames*. The desire for an easy-to-use package for interacting with FHIR servers and data is not new. For example, the fhircrackr [30] package was built to provide an interface for the automated extraction of FHIR data in the R programming language. In addition, several Python-based packages exist. The client-py [34] package has a query functionality and builds FHIR resources as object classes, but does not deal with *DataFrames*. Similarly, the fhir.resources [35] package builds object classes from the FHIR resources and validates them against the FHIR standard requirements, which can sometimes cause problems, as not all FHIR servers follow the standard strictly. Another package, fhir-py [36], performs asynchronous calls and allows for saving resources, but is also more focused on data retrieval from the FHIR server. Finally, fhirpack [37] has the same purpose as our package but with a different focus and structure in mind.

Three of the mentioned tools offer an interface for FHIR data extraction, but client-py and fhir-py are more focused on pure data extraction. In addition, the tools mentioned have no support for structured data export, e.g., into pandas *DataFrames*. Only fhirpack also tries to save the data in a structured way. However, this tool is more interested in an abstraction of the FHIR logic, and thus many FHIR mechanisms are not made accessible to the user.

The key features of our tool are:

• Easy to use, as the examples present on the project website can be easily customized to cover any use case.
• Works with all FHIR resources and does not need customization.
• The user has control over the attributes that will be extracted. They can either extract the entire resource or have multiple options to select which attributes are relevant.
• Supports FHIRPath [38], which is a standardized syntax similar to the one of JSONPath [39]. The relevant information can be selected by specifying the attribute path, or by enforcing conditions such as existence or equality.
• Combines clinical data as well as DICOM support (if a connection to a DICOMweb compliant imaging storage is available).
• Text mining support for medical documents.

**Fig. 2:**
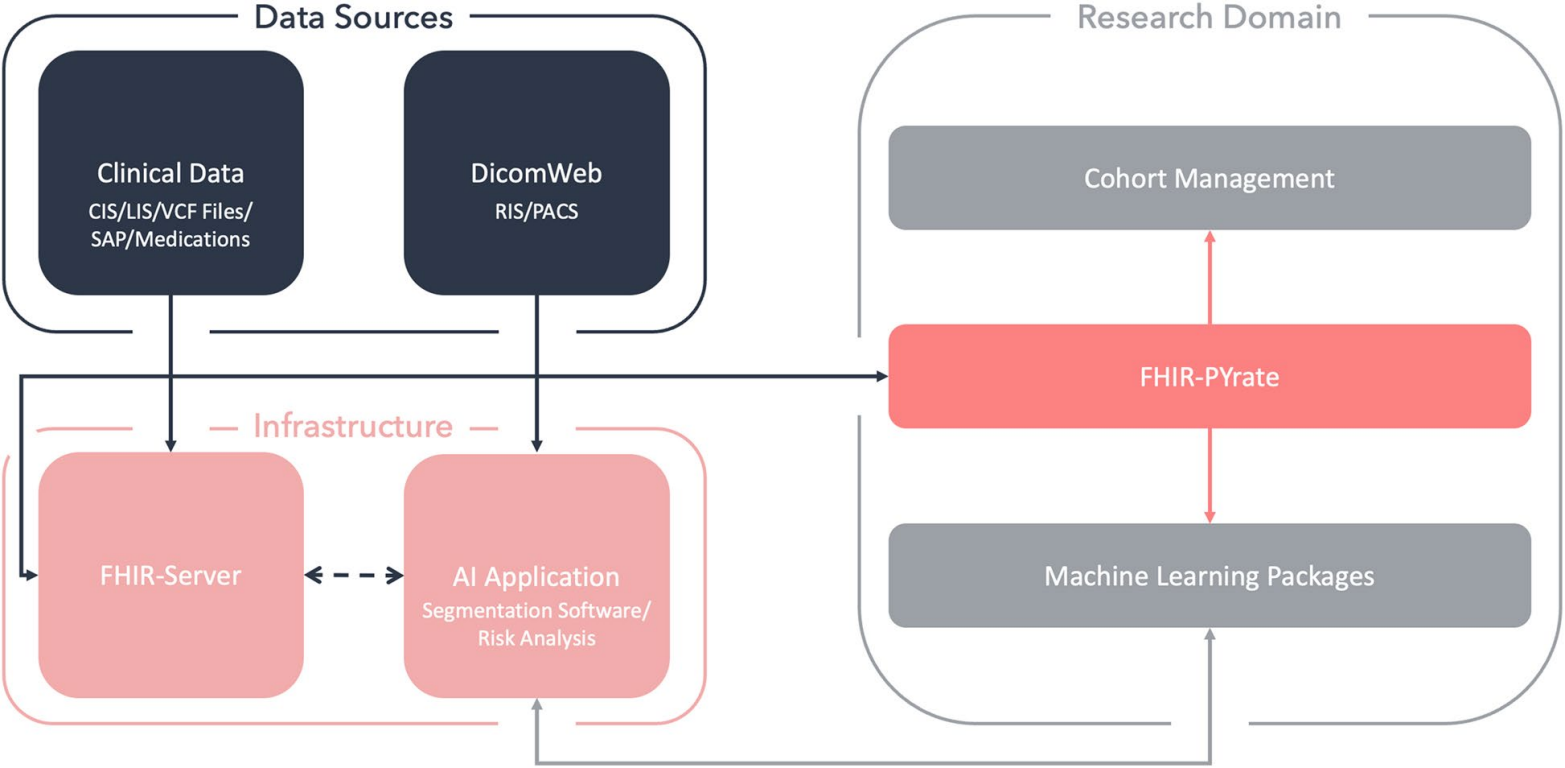
Overview of the tool’s role in the healthcare/machine learning picture. Presentation of an exemplary hospital infrastructure including multiple apps: a FHIR server, a DICOM Web capable PACS (or alternatively an app that handles the communication to the medical imaging storage), and various AI applications. Within the research domain, the FHIR-PYrate package handles the communication between the FHIR server and the PACS (DICOM Web). The package also helps with creating the data prerequisites needed for implementing machine learning solutions and for creating cohorts

The tool is supposed to integrate into a modern hospital's broader healthcare infrastructure, as described in Fig. 2. In this architecture, the clinical data comes from multiple sources, and can, for example, be transformed using ETL (*Extract, Transform and Load*) processes before being imported into the FHIR server. The clinical data may come from different systems, such as: LIS (*Laboratory Information System*), CIS (*Clinical Information System*), VCF (*Variant Call Format*) files, SAP (*System Analysis Program development*) and medication workflow solutions. The imaging data is stored in a RIS (*Radiology Information System*) or in a PACS (*Picture Archiving and Communication System*), which usually also have a DICOMweb interface [40]. The research domain is a separated system where the FHIR data is anonymized or pseudoanonymized, and can thus be used for cohort management of clinical studies and to build machine learning solutions. Using these, multiple AI (*Artificial Intelligence*) applications can be implemented for research purposes, for example, to compute automatic segmentations [41, 42], or for risk analysis [43]. Additionally, commercial AI software may also be included into the infrastructure [44], which can in turn be used to optimize the clinical workflows and for additional clinical studies.

The package is of aid for researchers to implement machine learning solutions and statistical analysis and for doctors to build cohorts of patients for studies. A cohort is a collection of patients that can be grouped according to a particular characteristic. A cohort can be built only with clinical data (e.g., Observation, Condition) but also with imaging data. For example, it is often the case that there is interest in collecting all patients based on a certain diagnosis or treatment. Then, for example, all CT scans of the abdomen could be collected and downloaded for these patients. This process is exactly one of the strengths of the package: Being able to build any cohort, regardless of the resource or even chain of resources. If the CT scans should also be downloaded for further analysis, it is also essential to have direct communication to the FHIR server and to a PACS, where the imaging studies are stored.

## Implementation

**Fig. 3:**
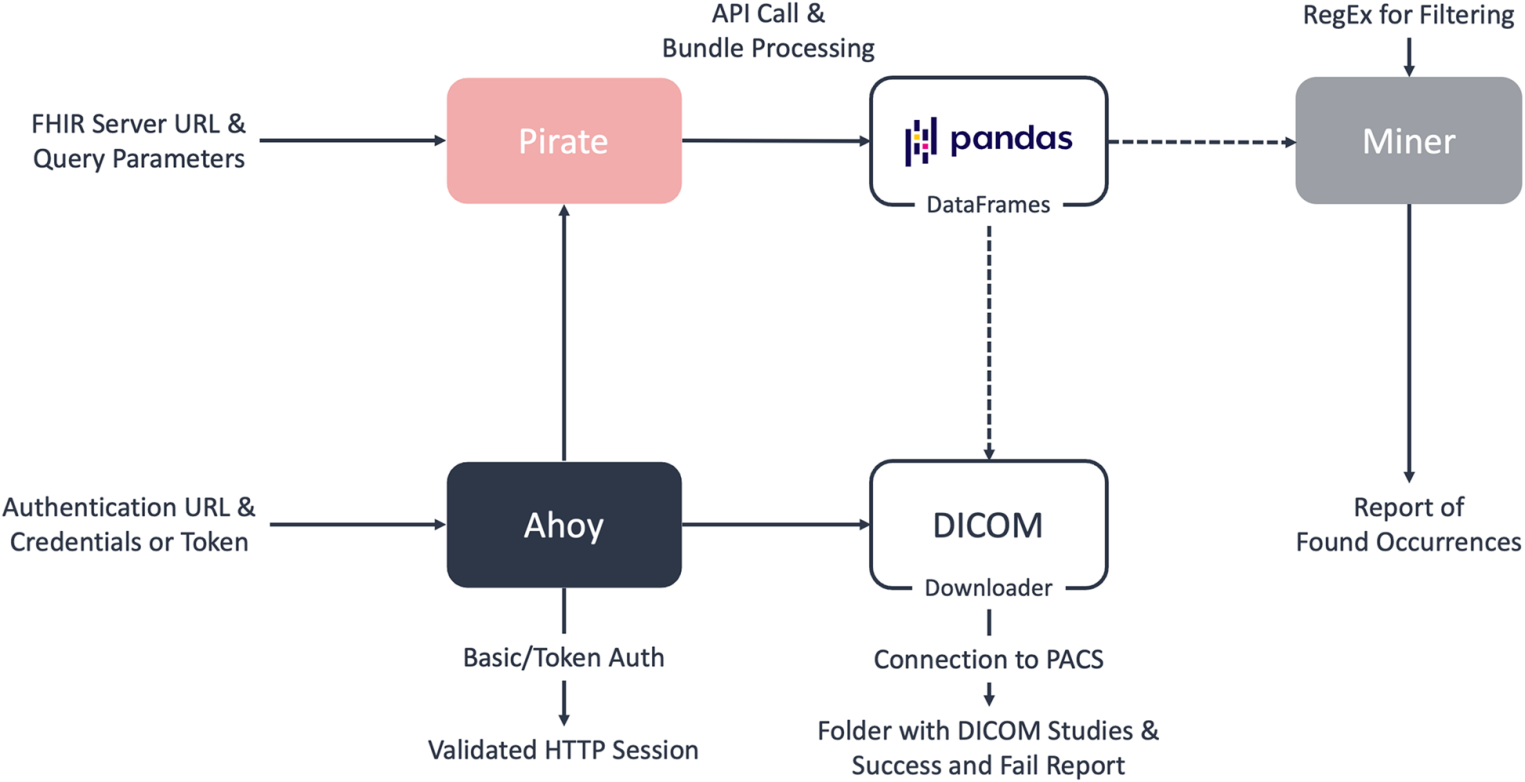
A schematic view of the package structure. The *Ahoy* class handles the user's authentication and creates an HTTP session, which the other classes will then use to interact with the FHIR server. The *Pirate* class handles the communication with the REST API, collects Bundles, and builds the *DataFrames*. The *Miner* class is to be used on a *DataFrame* (normally the result of a DiagnosticReport query), and it will output a report on whether a particular regular expression is present in a text, and in which particular parts of the documents. The *DICOMDownloader* class is used to download medical imaging scans in bulk and store information about them. The *Miner* and the *DICOMDownloader* class are connected with dashed lines, as they can be used as an optional step after retrieving the initial data

The package is open source and available under the following link: https://github.com/UMEssen/FHIR-PYrate. It can be installed with the pip Python package manager and all relevant information for installation and usage are reported on GitHub. The package is built on four main classes: *Ahoy*, *Pirate*, *DICOMDownloader*, and *Miner*, as presented in Fig. 3. In the following, we will describe their implementation and explain our stylistic choices. Most FHIR servers require authentication to access clinical data as a prerequisite for data extraction. The *Ahoy* class was implemented for this eventuality, supporting basic HTTP and token authentication. Its primary purpose is to create an HTTP object with the necessary headers for a later connection to a specific FHIR server. Additionally, this class ensures that the token stays up-to-date by either refreshing or reauthenticating the session.

### Pirate

**Fig. 4:**
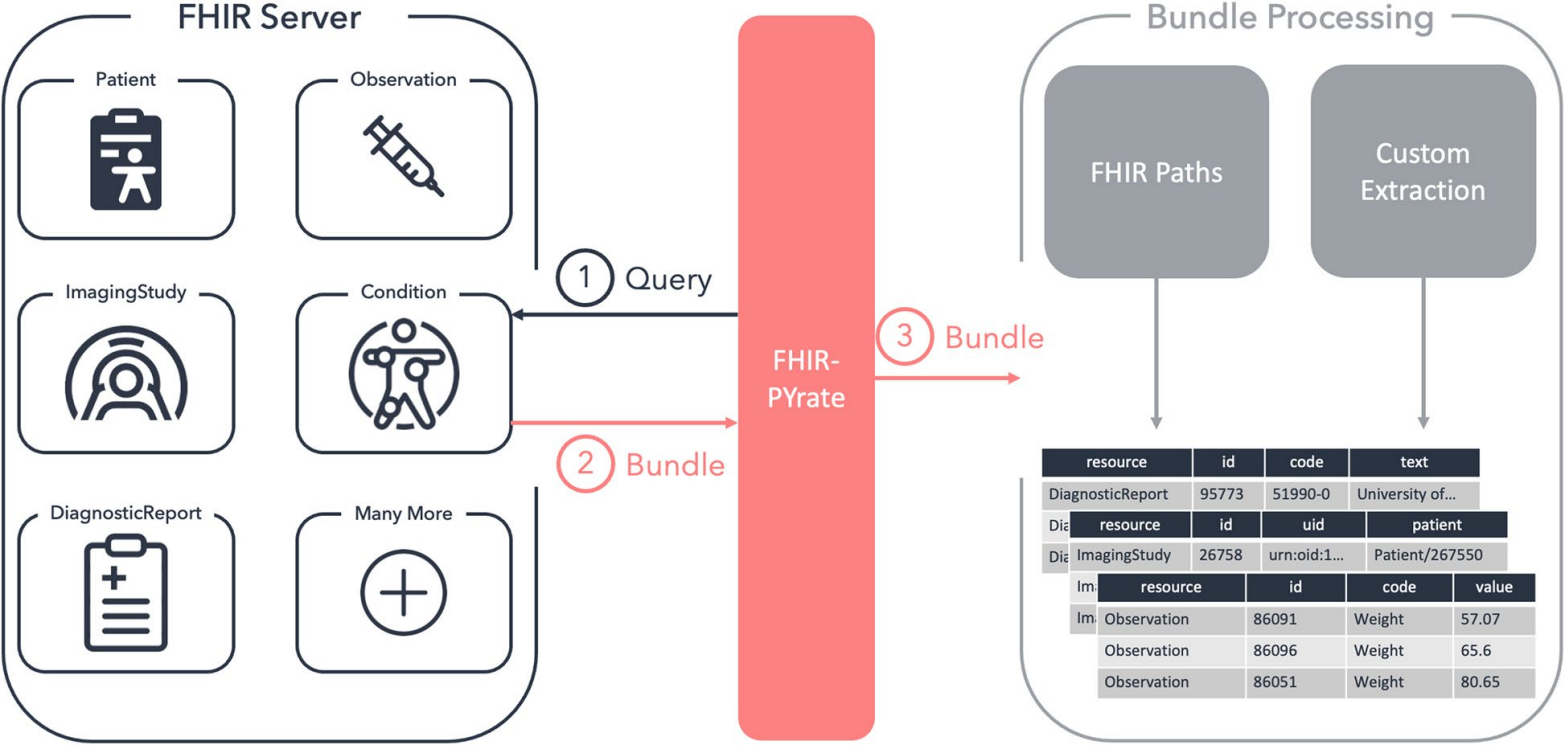
Schematic view of the process within the *Pirate* class. The *Pirate* class can be used with any existing resource, and data retrieval processes remain unchanged. In this figure, the querying process for the pictured resources is always the same: First, a query has to be defined and run, then, a FHIR Bundle is returned, and finally, the Bundle is transformed into a *DataFrame*

The *Pirate* class is the main interaction point for the data collection and handles all the communication with the FHIR server. The main idea of this class is to allow an easy collection and handling of clinical cohorts for researchers and doctors by using common data structures such as tabular data, which can, in turn, be converted to Excel files. In general, with an instance of the *Pirate* class, it is possible to access all FHIR resources (as shown in Fig. 4) and to use all implemented FHIR search parameters. The user has full control over the queries and can decide which operations should be done server-side by the FHIR server and which ones should be done client-side within the Python program. The Pirate class can handle all search parameters supported by the server, allowing for easy customization since each FHIR server may designate different parameters. Additionally, the user can also request a Bundle containing the edit history of a particular resource.

The functions contained in the *Pirate* class have one of three objectives:

1. **Request to** Bundle: They handle the communication with the FHIR server, run the query and return the FHIR resources in the form of a list of Python objects representing the Bundles.
2. Bundle **to** ***DataFrame***: They map the data in the obtained Bundles to *DataFrames*. In our schema, a row usually represents one resource.
3. **Facade functionality**: They combine the functionalities of 1 and 2 to make querying and obtaining *DataFrames* more convenient.

Additionally, the functions can either:

a run a query only according to the resource type and request parameters, or
b run a query according to the resource type, request parameters, and constraints given by an existing *DataFrame*.

The *Pirate* class has seven main data retrieval functions. The steal_bundles function takes the input request parameter and runs one single query on the FHIR server and collects the returned data as Python objects. The sail_through_search_space function uses concurrent processes to speed up the data retrieval. Usually, the user is interested in resources coming from a particular time frame. This function divides the desired time frame into as many time spans as there are processes available, and runs one query for each span. The trade_rows_for_bundles function also uses concurrent processes, but this time by spawning one process for each row of the input *DataFrame*. In fact, this function can be used when, for example, a cohort definition is already defined in a *DataFrame*, and we would like to retrieve information related to each single row. The function uses the given request parameters together with the constraints that are given by the *DataFrame* to build as many queries as there are rows, that are then run concurrently. The bundles_to_dataframe function takes the input of the previous functions and transforms it into a *DataFrame* by transforming the Bundles into rows. As per default, all FHIR attributes are stored in the final *DataFrame*, but some filtering mechanisms can also be used, which will be described later.

The steal_bundles_to_dataframe, sail_through_search_space_to_dataframe and trade_rows_for_dataframe are functions that combine their non-*DataFrame* variant with the bundles_to_dataframe function and perform both operations together. The trade_rows_for_dataframe function also has the option to add the original columns as columns to the final DataFrame to make further processing easier. A summary of the functions and their roles is presented in Table 1.

**Table 1:** Overview of the *Pirate* functions and of their capabilities. The functions of type 1 handle the conversion from query to Bundle, while the ones of type 2 build *DataFrames* from the Bundles. The functions of type 3 are facade functions that combine the functionalities of type 1 and 2 for convenience. Additionally, we have functions that only retrieve data according to the request parameters (type a), and functions that take a *DataFrame* as input and process all the rows (type b). Furthermore, we also support concurrent querying and computing for all functions but one, which is not parallelizable, as it only runs one query

Function	Type	Constraints on *DataFrame*	Parallel	
			**Queries**	***DataFrame* Building**
steal_bundles	1a. Request to Bundle	No	No	/
sail_through_search_space	1a. Request to Bundle	No	Yes	/
trade_rows_for_bundles	1b. Request to Bundle	Yes	Yes	/
bundles_to_dataframe	2. Bundle to *DataFrame*	/	/	Yes
steal_bundles_to_dataframe	3a. Facade (1 + 2)	No	No	Yes
sail_through_search_space_to_dataframe	3a. Facade (1 + 2)	No	Yes	Yes
trade_rows_for_dataframe	3b. Facade (1 + 2)	Yes	Yes	Yes

**Fig. 5:**
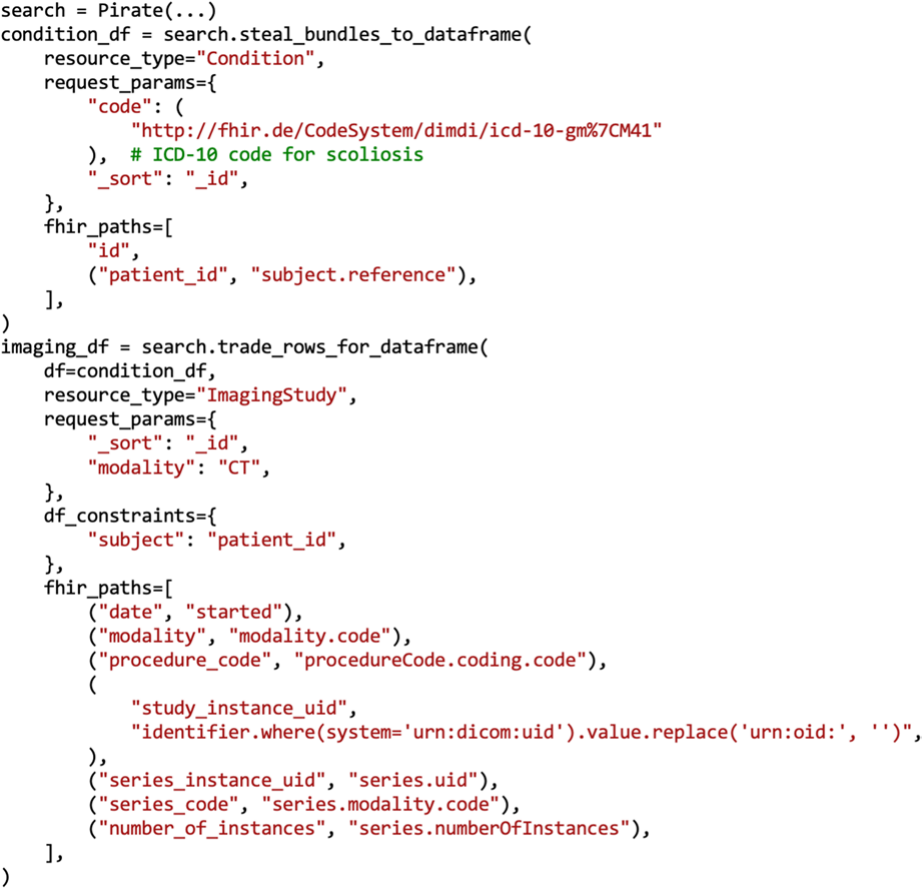
Retrieval of all CT studies for patients with scoliosis. Two FHIR-PYrate queries can be used to obtain all CT studies belonging to patients which suffer from scoliosis. The first query is for the Condition resource, while the second one is for the ImagingStudy resource. The request parameters for the FHIR server are specified using the request_params parameter. The df_constraints parameter is used to specify request parameters that should be constrained according to each row of the input *DataFrame*. The fhir_paths parameter selects which attribute of the resource should be returned

For example, a specific use case for the application of FHIR-PYrate would be the extraction of all CT scans from patients with a certain disease. One way to address this problem is to first find all Observation resources with a certain ICD (*International Classification of Diseases*) code [45], then use these resources to retrieve the Patient resources, and finally collect all the ImagingStudy resources for these patients. This can be achieved with two FHIR-PYrate queries, which are presented in Fig. 5.

The first query is of type 3a, as we just need to specify the kind of resource (Observation), and the required ICD code as a parameter. Each Observation has a reference to the corresponding Patient resource that has a diagnosis. The second query is of type 3b, and takes the kind of resource (ImagingStudy) and the *DataFrame* that we obtained from the previous query as input. Now, a query on ImagingStudy will be run for each row of the *DataFrame* of Observation resources, where the query’s Patient reference will be constrained on the subject of the Observation.

However, it often happens that the FHIR server contains multiple instances of the same patient, as they might be identified by multiple accounts on the hospital’s systems. If the server implements a heuristic that links the Patient resources with the Patient.link attribute (e.g. such as having the same name and date of birth assumes being the same person), then one additional query of type 3b on the *DataFrame* of Observation resources can be used to retrieve all the Patient resources.

**Fig. 6:**
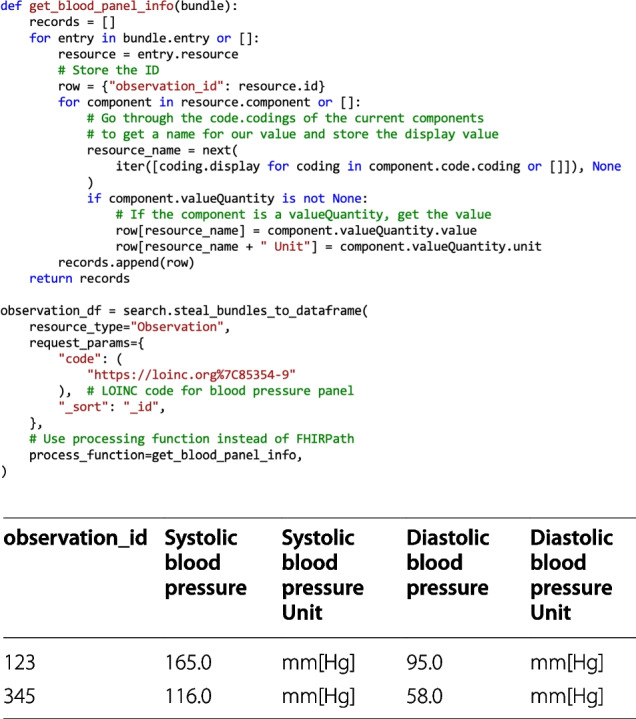
Using processing functions. The steal_bundles_to_dataframe retrieves the required Observation resources that contain information about the blood pressure panel. Then, the obtained bundles are transformed to rows by using the get_blood_panel_info function. This function iterates through the entries of a bundle and collects the resource IDs, and then iterates through the component attribute, which contains multiple pieces of information about the blood panel status, in this case, the systolic and the diastolic blood pressure. Each component attribute contains a display name (a natural language name of what is being evaluated), a quantity (the actual measured value) and a unit of measurement. For each piece of information, the display name becomes the column header, the quantity becomes the value, and the unit is stored in an additional column. The table below the figure presents an example output for this query

As we previously mentioned, FHIR resources can be arbitrarily complex, and thus it is important to implement mechanisms to filter only the needed information. The filtering is run while building the *DataFrames*, so all functions that have *DataFrames* as output support filtering. We offer two kinds of filtering, all Bundle-based:

1. Filter using FHIRPath: FHIRPath [38] is a standardized query syntax that accesses the desired attributes and performs simple operations such as replacing strings, simple mathematical operations, and checking for existence. The advantage of this is that it is rather easy to store any needed attribute with a small amount of code (as shown in Fig. 5). In this case, each row of the *DataFrame* represents one resource. We implement this feature with the help of fhirpath-py [46], a Python package built exactly for this purpose.
2. Filter using processing functions: Another option is to use a processing function, which can be given as input to the functions that transform the Bundles into *DataFrames*. These functions are always run for each Bundle and can implement any desired logic (example in Fig. 6). In this case, the *DataFrame* structure can be built arbitrarily, and each row does not necessarily represent one resource.

Another essential aspect is the possibility to run concurrent processes to more quickly process the queries. This, however, highly depends on the throughput of the FHIR server and on how many requests it can handle. The default number of processes is always one and can be increased freely according to the server’s capabilities.

**Fig. 7:**
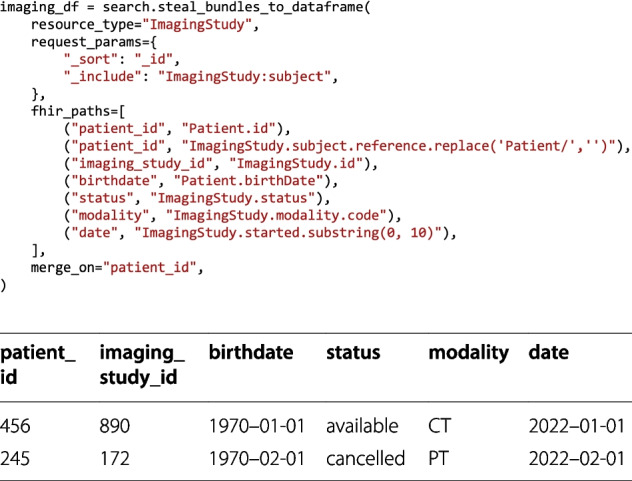
Including secondary resources. ImagingStudy resources also contain a reference to the Patient resource they belong to, and using the _include parameter the corresponding Patient resources can be imported in the output Bundle. The attributes that should be added to the final *DataFrame* can be specified with the fhir_paths parameter, where it is also possible to specify from which resource the attribute should come from, to ensure clarity. Usually, one row of the output *DataFrame* represents one entry of a Bundle. By specifying the merge_on parameter, the rows which have the same patient_id are merged, producing a *DataFrame* similar to the table below the figure

We implement three kinds of parallelization. First, whenever multiple queries are generated from one *DataFrame* (functions of type a), this can be run in parallel by setting the number of desired processes. Second, the conversion from Bundle to *DataFrame* can also be performed concurrently. Last, if the amount of data to retrieve concerns a vast time span, the time span can be broken into as many time frames as there are processes. For all facade functions supporting multiprocessing, the user can decide whether they want to run the queries first and then build the output *DataFrame*, or whether they want to alternate between running queries and transforming them using the filtering functions. A direct transformation after each bundle retrieval ensures that if a data point does not apply to the filtering schema, the run will stop. If the bundles are retrieved first and then transformed, any errors will occur at the very end. Another notable feature is the possibility to merge information from multiple rows automatically after running a query. This may happen when a query includes resources of another type in the result. For example, a query on ImagingStudy may also want to return the Patient resources (as in Fig. 7), such that it is possible to process the studies and the patients together. In this case, since we usually expect one row to represent one resource, we would have rows corresponding to ImagingStudy resources, and rows corresponding to Patient resources. By specifying the merge_on flag with the name of the column that should be used for merging resources (in this case, with the same Patient ID), we obtain a *DataFrame* where each row represents an ImagingStudy and that has all the needed information about the patient.

### Miner

Medical findings are often stored in an unstructured manner, especially as free text. Thus, whenever a piece of information is needed from a document, it has to be found manually among all existing documents. Increasingly powerful NLP (*Natural Language Processing*) models have currently been used for named entity recognition to solve this exact problem [47–49]. However, they usually need some ground truth, are not available for all languages, and they must undergo stringent validation processes to be admitted into the clinical routine. The *Miner* class is supposed to aid NLP pipelines by identifying which documents are possibly relevant to a particular research question.

**Fig. 8:**
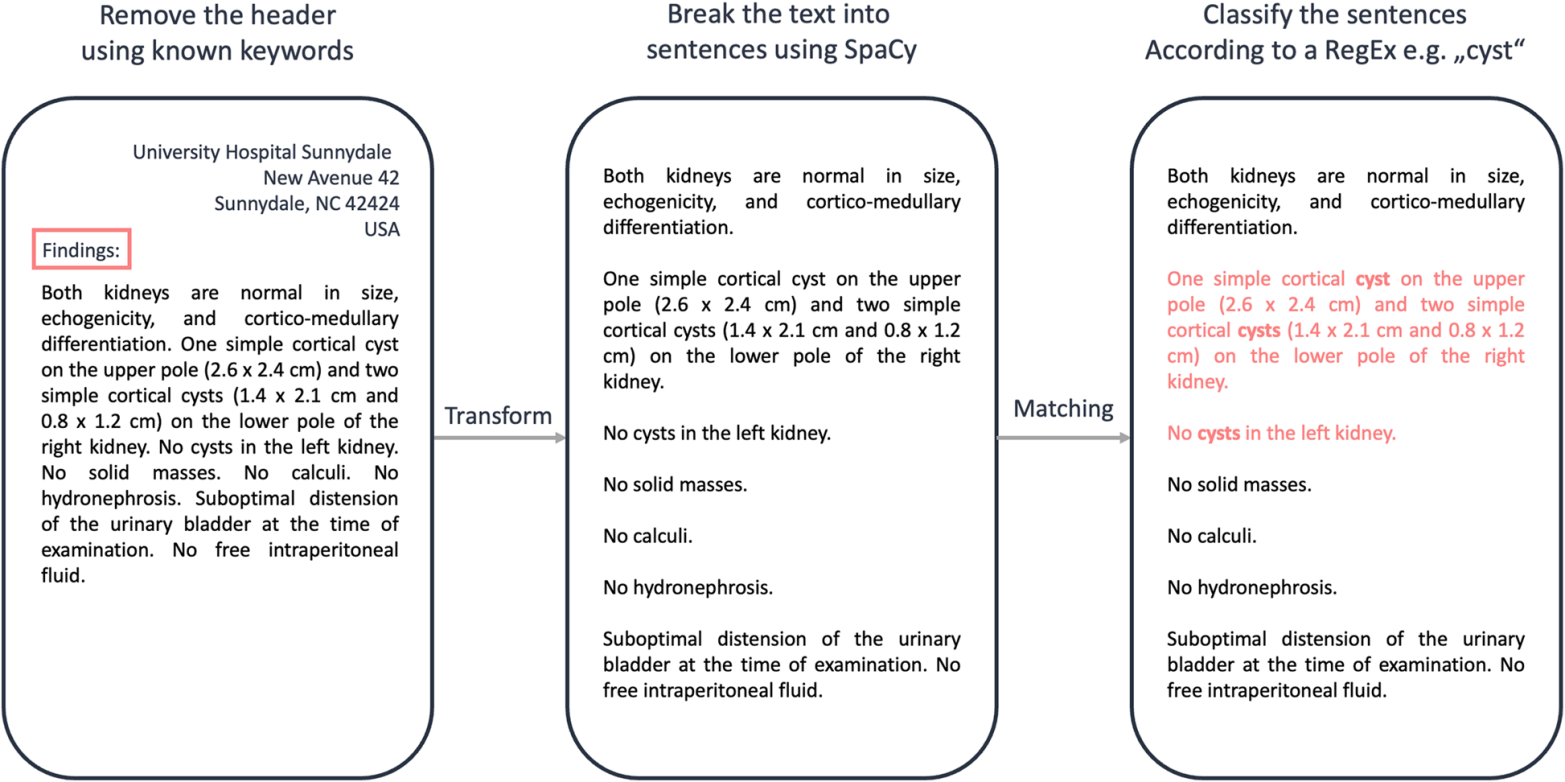
An overview of the process to identify whether a text contains relevant sentences using the *Miner* class. First, if the document has a specific structure, all the information before a known keyword (e.g., “Findings”) is removed. Then, the sentences are identified using the SpaCy library. For each sentence, the input regular expression is matched against the text and if the sentiment of the sentence is not negative, the sentence is considered a match

The main idea of the class is to take a *DataFrame* containing texts and find out whether a user-given regular expression is present in the document, as presented in Fig. 8. This is done by first converting the text into sentences and then checking whether the regular expression is present in each sentence. As a result, the user is given a summary of the locations of the searched expression, and the number of viable instances. Additionally, it is also possible to give a secondary regular expression as input, and in such cases the result is the sentences that contain the first regular expression and do not contain the secondary one. This process may be useful to find some particular keyword (e.g., sarcoma), but at the same time to remove specific cases that are not interesting for our research question (e.g., osteosarcoma). The sentences are created using the well-known NLP library SpaCy [50], while the regular expression matching is done with the Python standard library re [51].

**Fig. 9:**
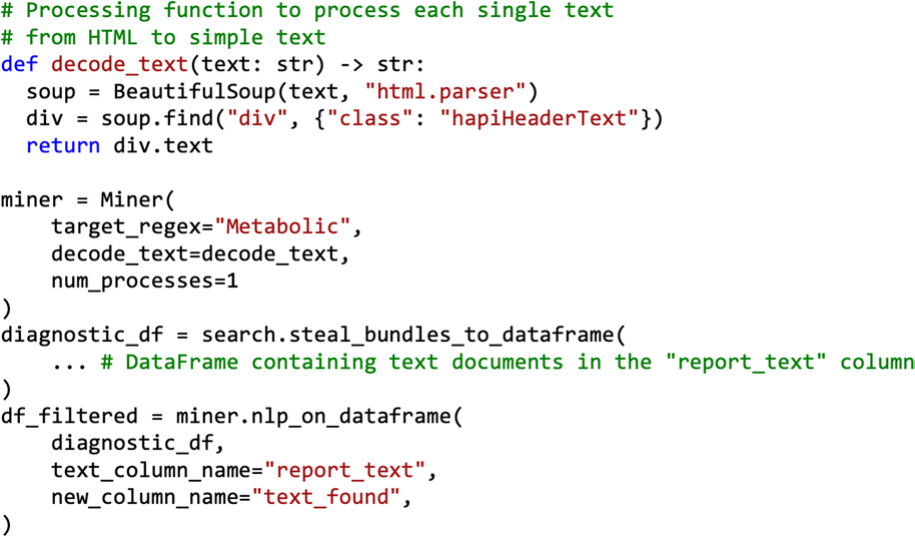
Example *Miner *Query. The *Miner* class is first initialized, then, a *DataFrame* containing the text documents in a specified column is processed using the nlp_on_dataframe function. However, the data may not be stored as readable text on the FHIR server (e.g. may be stored as HTML, encoded). For this purpose, a processing function to preprocess the text may be specified, in this case, decode_text

In many cases, clinical documents have a specific structure. For example, documents may always have a header, and the relevant part of the text may always start with the word “Findings”. In such cases, it is beneficial to remove the header, and this can be done by giving these specific keywords as input to the ​​nlp_on_dataframe function (Fig. 9). This class function handles the entire described process, and if requested, the rows of the *DataFrame* can also be examined concurrently using multiple processes.

### DICOMDownloader

In recent years, deep learning for medical imaging has become a very active field of research, resulting in a great need for direct and automatable access to image data by data scientists. Most imaging studies conducted within the clinical routine are stored in a PACS using the DICOM format. In our system, the DICOM studies are referenced as resources on a DICOMweb endpoint by ImagingStudy resources on the FHIR server. Each ImagingStudy references a DICOM study, which can reference multiple series (i.e., the actual scans). In the DICOM standard, each scan has a SeriesInstanceUID and a StudyInstanceUID.

The *DICOMDownloader* class aims at automating the downloading process of DICOM studies or specific series. As a prerequisite, we need a pandas *DataFrame* containing the StudyInstanceUID of the study we want to download. In the event that only certain series are to be downloaded, a SeriesInstanceUID can also be supplied. Once the cohort of series has been identified, a call to download_data_from_dataframe stores the downloaded series in the desired folder. Optionally, the series may be stored according to a hierarchical folder structure, which reduces the number of files per folder and may be beneficial to avoid overloading the file system.

**Fig. 10:**
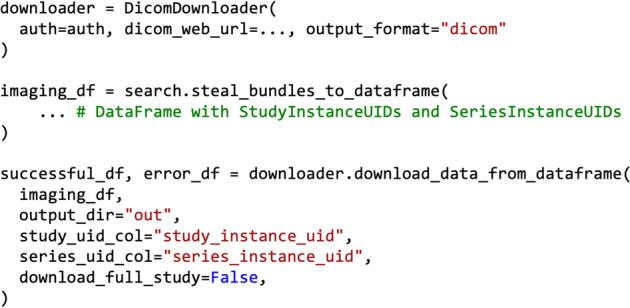
Example *DICOMDownloader* Query. The *DICOMDownloader* class is initialized with the desired output format, and the data is downloaded to the desired output directory using the download_data_from_dataframe function

**Fig. 11:**
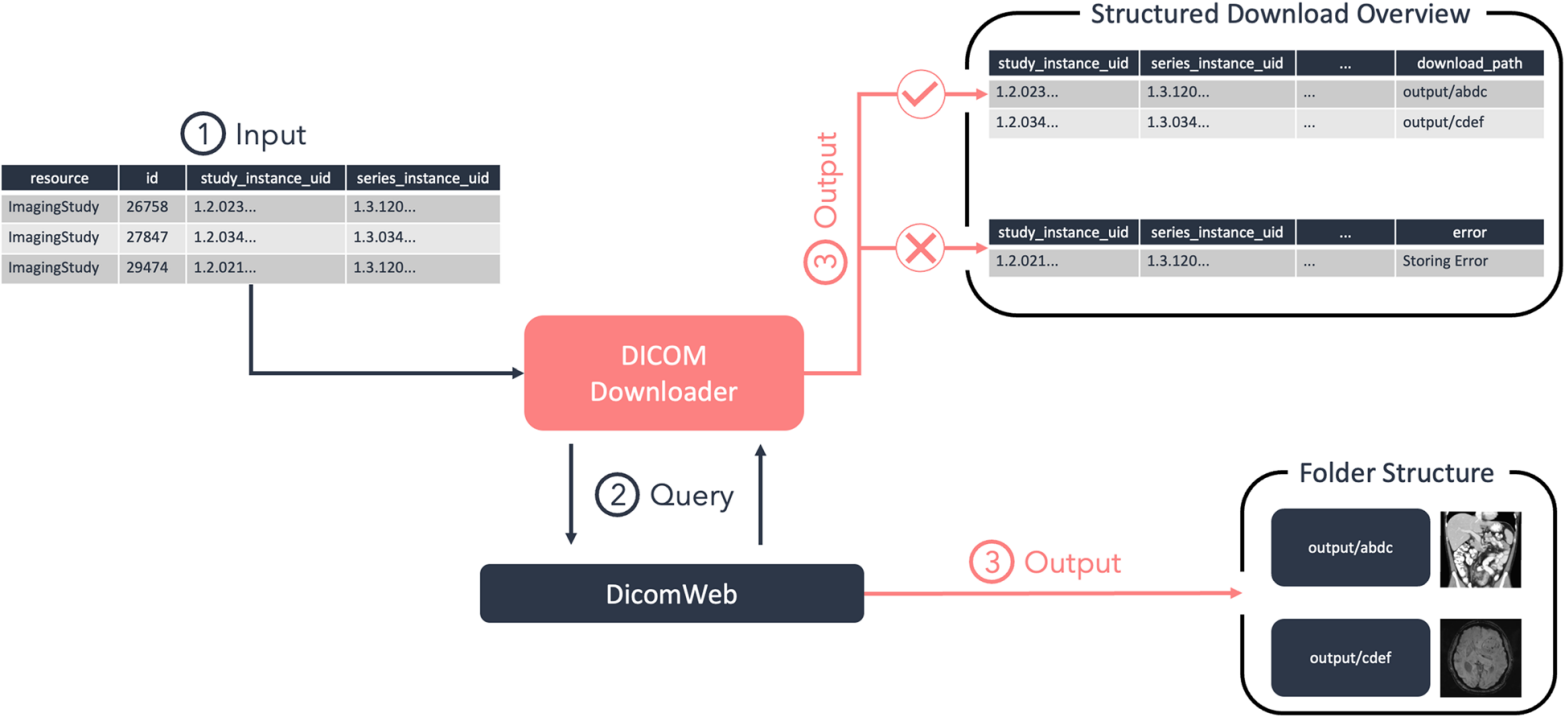
An overview of the download of DICOM studies using the *DICOMDownloader*. A collection of StudyInstanceUID and SeriesInstanceUID is given as input to the DICOMDownloader (1), which uses them to communicate with a DICOMweb instance (2) and stores the results in a predefined folder (3). Additionally, two *DataFrames* are returned (3). The first one contains a list of all successfully downloaded series, while the second one has a list of all the failed series and the kind of error that was produced

The communication with a PACS is handled by the DicomWebClient package [52]. This assumes that the PACS is able to handle the DICOMweb standard [40]. Once the series have been downloaded, the download_data_from_dataframe function returns two *DataFrames* (Fig. 10), one containing the series that could be successfully downloaded and the name of the folder where they are stored, and the other containing the IDs of the series that failed, and the error that caused them to fail. This process is also depicted in Fig. 11.

## Results

To demonstrate the practical applicability of this package, a real-world scenario is presented to analyze the prognostic significance of routine CT imaging and clinical data in breast cancer patients with pulmonary metastases. Since triple negative breast cancer is known to have a poor prognosis [53], ER (*Estrogen Receptor*), PR (*Progesterone Receptor*), and HER-2 (*Human Epidermal growth factor Receptor 2*) are highly relevant markers. In this example, a survival analysis will be performed using a deep learning model with the CT of the thorax, age, TNM stage, ER, PR, and HER-2 status of the selected patients.

**Fig. 12:**
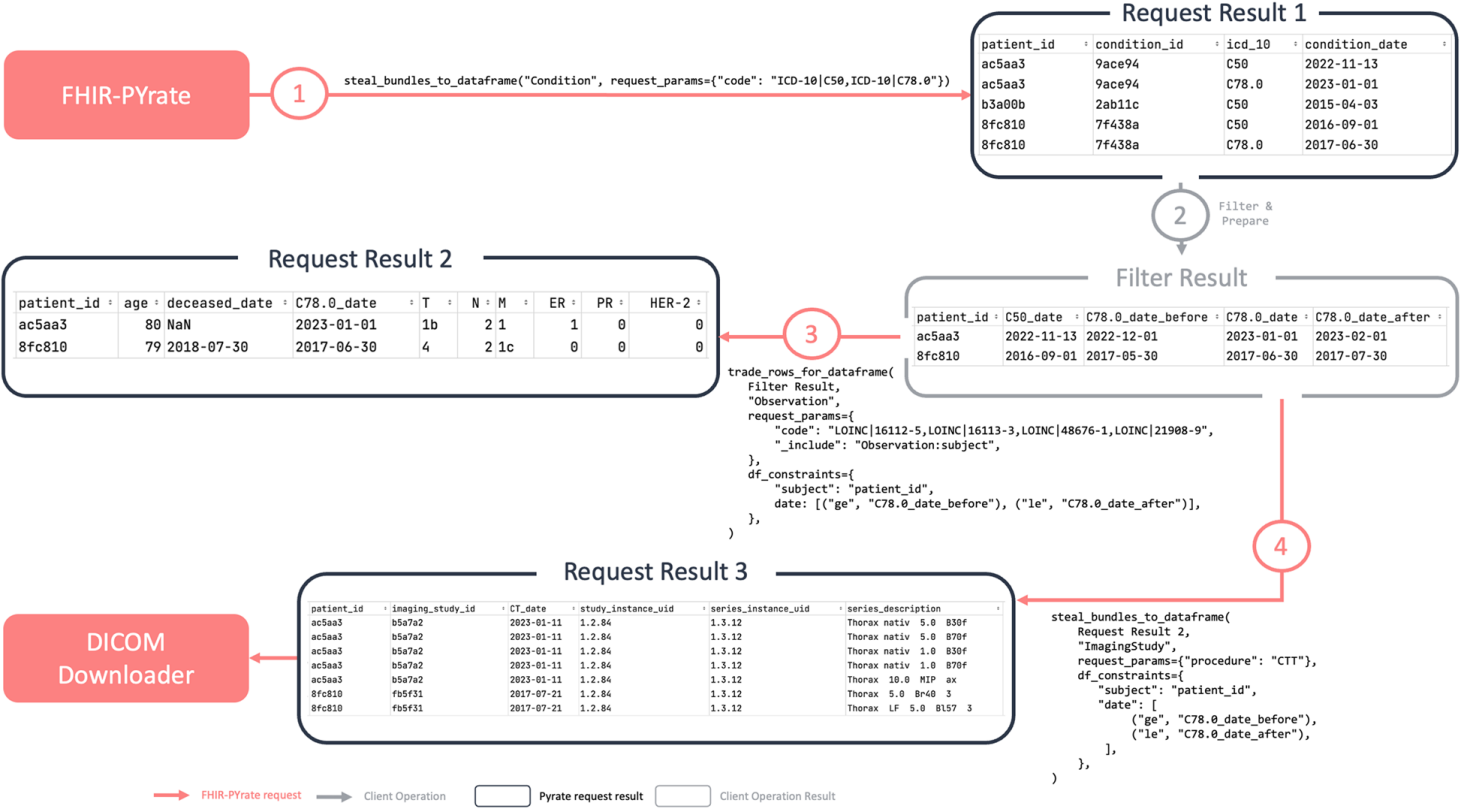
Data collection using FHIR-PYrate for breast cancer patients. The process starts by collecting patients with specific ICD-10 codes (1), the results are filtered (2), and then the clinical (3) and imaging data (4) is retrieved. The resulting *DataFrames* can also be merged to obtain the complete cohort information. The code retrieval is simplified by omitting the URL of each system (i.e., LOINC instead of "http://loinc.org")

The first step is to find all patients with metastatic breast cancer. The patients can be identified using the Condition resource with the ICD-10 code “C50” for breast cancer, and “C78.0” for pulmonary metastases, and by selecting the patients that have both. For the remaining patients, some demographic information is retrieved, such as age and date of death. For the survival analysis, the samples are going to be censored if the patient has died, and uncensored otherwise. The time to death will be specified as the number of days between the diagnosis date of the metastases and the death of the patient, or a specific date to be chosen as the end of the study. Additionally, the clinical data is retrieved using the Observation resource and the respective LOINC (*Logical Observation Identifiers Names and Codes*) codes [54] and by checking that the date of the Observation is at most 30 days before or after the diagnosis of the metastases. The resulting table contains the ER (LOINC code “16,112–5”), PR (LOINC code “16,113–3”) and HER-2 (LOINC code “48,676–1”) markers and the TNM stadium (LOINC code “21,908–9”) for the selected patients. Similarly, ImagingStudy resources referencing CTs of the thorax are retrieved using the same date constraints and are downloaded using the *DICOMDownloader*. This process is outlined in Fig. 12. It is important to notice that the available data and the use of particular codes (e.g., LOINC, ICD-10) may depend on the FHIR server and on the clinical data available at an institution. Additionally, some FHIR servers do not implement all search parameters, and there might be different approaches to obtain the same results.

The patient cohort can be then divided into a train and a test set, and a deep learning model can be trained to predict the survival of the patients using a Cox regression. Previous studies have already successfully used deep learning methods for survival analysis [55], and could be extended to also consider imaging information.

The described process can be used for any use case requiring both clinical and imaging data, and could also be extended to NLP by also retrieving the DiagnosticReport relevant to the use case.

## Discussion

The tool offers a connection between the well-structured and complex FHIR standard and the field of data science, which is dominated by the use of tabular data. Its *DataFrame* output delivers an easy-to-understand, simplified overview of the FHIR resources and can be used more easily for statistical analysis and machine learning. The filtering capabilities also ensure that the user is only provided with the data they need and that the output is not just a dump of the FHIR resources. Furthermore, the filtering allows the user to perform simple operations on the data (e.g., replacement, splitting) such that the final *DataFrame* does not require further modification. An important criterion in the design of our package was not—by means of abstraction—to keep the user away from the FHIR standard, which in our view is well designed, very intuitive and at the same time powerful, but rather to allow the user efficient access to the data contained in a FHIR server while retaining full control over the underlying queries and data structures. The package is intended as a scripting tool, but a more user-friendly graphical interface to be used with the tool as its core is already being planned. In this regard, the tool can also be used to build dashboards and monitors to display important FHIR metrics and data, and that can actively be used in patient care.

## Conclusions

In this work, we presented FHIR-PYrate, a Python package for extracting and collecting all sorts of clinical data from FHIR servers. The proposed tool is essential for researchers, but also for technology-affine doctors, to rapidly build research cohorts and to combine clinical as well as imaging data. The main *Pirate* class handles the communication with the FHIR server and can be used for any resource type, which makes the creation of any dataset straightforward. Additionally, we presented two additional classes: The *Miner* class to filter attributes according to regular expressions and the *DICOMDownloader* to download imaging studies directly from a PACS. With its simplicity and standardized mechanisms, the FHIR-PYrate uses all the advantages of the FHIR standard and combines it into one Python based API.

In future work, we plan to further analyze and test this package with FHIR servers from multiple institutions. Additionally, we aim at simplifying and streamlining the main package API to make it more accessible to users with various levels of programming experience. The newest developments will be posted on the GitHub page of the package (https://github.com/UMEssen/FHIR-PYrate).

## Availability and requirements

**Project name**: FHIR-PYrate.

**Project home page**: https://doi.org/10.5281/zenodo.7025226

**Operating system(s)**: Platform independent.

**Programming language**: Python.

**Other requirements**: Python 3.7 or higher (for version 0.1.0), Python 3.8 or higher.

**License**: MIT.

**Any restrictions to use by non-academics**: None.

## Data Availability

Not applicable.

## Abbreviations

AI: Artificial Intelligence
API: Application Programming Interface
CDA: Clinical Document Architecture
CIS: Clinical Information System
CT: Computed Tomography
DICOM: Digital Imaging and Communications in Medicine
EHR: Electronic Health Records
ER: Estrogen Receptor
ETL: Extract, Transform and Load
FHIR: Fast Healthcare Interoperability Resources
HER-2: Human Epidermal growth factor Receptor 2
HL7: Heath Level Seven
ICD: International Classification of Diseases
LIS: Laboratory Information System
LOINC: Logical Observation Identifiers Names and Codes
ONC: Office of the National Coordinator for Health Information Technology
MRI: Magnetic Resonance Imaging
PACS: Picture Archiving and Communication System
PR: Progesterone Receptor
REST: REpresentational State Transfer
RIS: Radiology Information System
SAP: System Analysis Program development
VCF: Variant Call Format
